# The Therapeutic Potential of Photoimmunotherapy as a Safe, Effective and Non‐Toxic Treatment Option for Superficial Triple Negative Breast Cancer

**DOI:** 10.1002/cam4.71255

**Published:** 2025-09-19

**Authors:** Fleury Augustin Nsole Biteghe, Neelakshi Mungra, Zaria Malindi, Nyangone Ekome Toung Chalomie, Ketum Ateh Stanislas, Sayeda Yasmin‐Karim, G. Mike Makrigiorgos, Fallon Ester Chipidza, Olusiji Alex Akinrinmade, Srinivas Sridhar, Stefan Barth, Wilfred Ngwa

**Affiliations:** ^1^ Department of Chemistry and Chemical Biology College of Science, Northeastern University Boston Massachusetts USA; ^2^ Centre for Immunity and Immunotherapies Seattle Children's Research Institute Seattle Washington USA; ^3^ Faculty of Health Sciences, Laser Research Centre University of Johannesburg Johannesburg South Africa; ^4^ Medical Biotechnology and Immunotherapy Research Unit, Institute of Infectious Disease and Molecular Medicine University of Cape Town Cape Town South Africa; ^5^ Biopharmaceutical Training Laboratory (BATL), College of Professional Studies Northeastern University Burlington Massachusetts USA; ^6^ Johns Hopkins School of Medicine Baltimore Maryland USA; ^7^ Department of Radiation Oncology Dana Farber Cancer Institute Boston Massachusetts USA; ^8^ Department of Radiation Oncology Brigham and Women's Hospital Boston Massachusetts USA; ^9^ Department of Radiation Oncology Harvard Medical School Boston Massachusetts USA; ^10^ Department of Molecular Pharmacology Albert Einstein College of Medicine Bronx New York USA; ^11^ College of Engineering Northeastern University Boston Massachusetts USA; ^12^ Faculty of Health Sciences, Department of Integrative Biomedical Sciences, South African Research Chair in Cancer Biotechnology University of Cape Town Cape Town South Africa; ^13^ Sidney Kimmel Comprehensive Cancer Center, School of Medicine Johns Hopkins University Baltimore Maryland USA

**Keywords:** antibody drug conjugate, antibody photoconjugate, immunogenic cell death, photoimmunotherapy, triple‐negative breast cancer

## Abstract

Triple‐negative breast cancer (TNBC) is the most aggressive breast cancer subtype, lacking estrogen (ER), progesterone (PR), and human epidermal growth factor receptor (HER‐2) expression. It disproportionately affects women of African descent and has a poor clinical prognosis attributed to its acute heterogeneity, thereby causing elevated mortality rates. Due to the lack of well‐defined molecular targets in TNBC, treatment relies heavily on a trimodality approach (surgery, radiotherapy, and chemotherapy), despite growing evidence of adverse effects and disease relapses. Therefore, there is an urgency to identify targetable aberrations for more effective approaches capable of selectively detecting and killing targeted cells while sparing healthy tissues. The emergence of monoclonal antibodies (mAbs) targeting tumor‐associated antigens (TAAs), which can be used as a carrier to deliver highly cytotoxic drugs, raised hopes that antibody‐drug conjugates (ADCs) might solve the toxicity‐therapy challenge by shifting the balance more toward beneficial therapeutic efficacy. Despite their therapeutic benefits, their clinical translation is limited by key developmental barriers, including immune‐related adverse events. To address these limitations, a novel approach using antibody‐photoconjugates (APCs) was developed for photoimmunotherapy (PIT) applications, whereby local exposure to near‐infrared (NIR) light induces targeted phototoxic damage, culminating in apoptotic, necrotic, and immunogenic cell death (ICD) with minimal toxicities. Therefore, this review highlights the potential of PIT as an inherently safer and efficient light‐dependent therapeutic option for treating TNBC.

**Trial Registration:** NCT06449222

## 1 Introduction

### 1.1 Overview of Triple‐Negative Breast Cancer

Breast cancer (BC) remains the most prevalent cancer among North American women, claiming over 42,250 lives annually [1]. Globally, about 1 million new BC cases are diagnosed each year, with more than 170,000 exhibiting the triple‐negative phenotype [2]. Triple‐negative breast cancer (TNBC), the most aggressive BC subtype, comprises 15%–20% of cases [3, 4, 5, 6] and is defined by the lack of estrogen receptor (ER), progesterone receptor (PR), and HER‐2 expression [3, 4, 5, 6, 7, 8, 9, 10]. Its intrinsic heterogeneity and absence of clear molecular targets pose significant treatment challenges [11, 12].

Even with trimodality therapy (surgery, radiotherapy, and chemotherapy), up to 50% of early‐stage (I–III) TNBC patients relapse, and 37% die within five years of surgery [13, 14]. TNBC also disproportionately affects women of African ancestry, especially premenopausal women, though the reasons—whether genetic, environmental, or both—remain unclear [15, 16, 17, 18]. Risk factors include higher parity, younger age at first pregnancy, and shorter breastfeeding duration [19]. As Brewster et al. emphasize, multinational collaboration is urgently needed to unravel TNBC's causes, prevention, and treatment, particularly for women of African descent [20]. Given these challenges, researchers increasingly recognize the imperative to decode this complex disease. Routine screening methods like mammography and ultrasound remain inadequate for reliable TNBC detection. Dent et al. demonstrated that clinical examination identifies TNBC tumors more effectively than imaging modalities [21]. This aligns with observations that TNBCs frequently emerge as interval cancers between mammograms, likely due to their rapid growth rate or variations in breast tissue density [2, 22].

TNBC therapeutic options are limited compared to hormone receptor‐positive or HER‐2‐positive breast cancers, and the absence of specific guidelines necessitates reliance on conventional chemotherapy [18]. Metastatic TNBC is typically treated with chemotherapy regimens containing taxanes, anthracyclines, capecitabine, or ixabepilone [23]. Paradoxically, while TNBCs often show higher initial chemotherapy sensitivity than other subtypes [12, 13], responses are frequently short‐lived, as evidenced by retrospective analysis of 111 monotherapy and combination therapy cases [24]. Moreover, relapses commonly occurring in visceral organs, bones, and the central nervous system remain a major challenge [13, 25, 26].

In response to these challenges, immunotherapy research has prioritized the identification of actionable molecular targets in TNBC. Current investigational strategies target multiple pathways, including angiogenesis inhibitors, PARP1, p53 signaling effectors (PI3K/Akt/mTOR, Chk1, Hsp90), epigenetic regulators (HDAC), cell cycle proteins (CDK, Nutlins), and growth factor receptors (EGFR, FGFR) [27, 28, 29, 30]. Notably, despite extensive research, no targeted therapy has yet demonstrated consistent clinical efficacy for TNBC [30]. The advent of precision oncology, however, is enabling more systematic identification of disease‐driving transmembrane receptors, accelerating the development of both targeted therapies and predictive biomarkers for TNBC patients [31, 32].

EGFR, a transmembrane tyrosine kinase receptor overexpressed in more than 50% of TNBC cases, has been widely implicated in treatment failure [9, 33, 34]. Structurally, EGFR contains three domains: extracellular ligand‐binding, transmembrane, and intracellular tyrosine kinase [33, 35]. Upon ligand‐induced dimerization (homo‐ or heterodimerization), the receptor undergoes autophosphorylation, activating downstream pathways (RAS/MAPK and PI3K/Akt) that regulate cancer‐promoting and drug‐resistant gene expression [33, 35, 36]. Considering what precedes, immunotherapy using the US Food and Drug Administration (FDA)‐approved monoclonal antibodies (mAb) such as cetuximab and panitumumab to target EGFR was developed and viewed as a promising therapeutic option [9, 33, 34, 37, 38, 39, 40]. However, its therapeutic benefit in treating TNBC has yet to be demonstrated, as it can only achieve significant potency when combined with systemic chemotherapy and tyrosine kinase inhibitors (TKIs) [9, 33, 34, 37, 38, 39, 40]. Similarly, trastuzumab, a mAb against human epidermal growth factor receptor‐2 (HER‐2), was for the first time (1998) clinically approved to treat non‐TNBC HER‐2 positive breast cancer patients having completed chemotherapy [41]. Despite these promising results, this mAb suffered from efficacy problems, as more than 70% of the responders progressed into disease metastases a year post‐treatment [41]. These suboptimal therapeutic effects, compromising tumor regression, were partly attributed to a therapeutic resistant TNBC sub‐population overexpressing CD44 receptors [42, 43]. These recalcitrant TNBC cells have been reported to possess stem cell‐like properties, including self‐renewal, equipping them with the ability to recapitulate the entire parental tumor phenotype [42, 43]. Moreover, their resistance to chemotherapeutic and immunotherapeutic treatments has widely been documented and associated with aberrant expression of multidrug resistance (MDR) proteins, DNA repair, don't eat me signal (CD47), detoxifying enzymes, and immune checkpoint ligand (Program Death Ligand 1: PDL‐1) respectively [43, 44, 45, 46, 47, 48, 49, 50]. Based on these premises, achieving precision cancer medicine through the development of targeted immunotherapy using antibody drug conjugates (ADC) was developed [7, 50, 51].

These ADCs are considered homing missile immunoconjugates in virtue of their mAb capacity to selectively bind and release cytotoxic payloads within targeted tumor cells aberrantly expressing cognate tumor‐associated antigen (TAA) [7, 35, 50, 51, 52]. Lately, many ADCs have demonstrated significant potency against treatment‐refractory cancers, resulting in their FDA approval [53]. These ADCs include e.g., Kadcycla (ado‐trastuzumab emtansine) and Enhertu (trastuzumab deruxtecan), two anti‐HER‐2 mAbs respectively conjugated to maytansinoid (DM1: microtubule inhibitor) and camptothecin (topoisomerase I inhibitor) to treat locally advanced and metastatic breast cancer [53]. Additionally, troveldy sacituzumab govitecan, an anti‐trophoblast antigen 2 (TROP‐2) mAb conjugated to camptothecin was recently approved by the FDA (April 2021) to treat locally advanced and metastatic TROP‐2‐expressing TNBC patients [53]. Despite these significant advances, managing ADCs' immune‐related adverse events while balancing efficacy, remains a challenge.

Efforts to circumvent these undesired effects led to the combination of immunotherapy with new adjunctive therapeutic approaches such as photodynamic therapy (PDT), with proven success in treating breast and head and neck cancers [54, 55]. PDT is an FDA‐approved anticancer modality using a light‐activated compound (PS) to induce phototoxic effects damaging tumor vasculature and inducing tumor destruction through the activation of apoptosis, necrosis, and immunogenic cell death (ICD) [56, 57, 58, 59, 60]. However, PDT efficacy might be compromised by many factors, including poor therapeutic light penetration within tumor tissue; low levels of molecular oxygen in hypoxic tumors; and passive diffusion of PS in normal tissues, causing life‐threatening side effects. To bypass this lack of specificity and efficacy, photoimmunotherapy (PIT) was developed as a targeted PDT version. PIT is a form of light‐dependent immunotherapy using a photostable and water‐soluble phthalocyanine dye, IRDye700DX (IR700) as a photosensitizer (PS) and a mAb as the targeted moiety [52, 61, 62]. These APCs can specifically bind to target cancer, immunosuppressive, or stromal cells and generate death‐inducing amounts of reactive oxygen species (ROS) when exposed to optimal therapeutic light (690 nm) [52, 61, 62]. Therefore, these PIT‐induced phototoxic effects will ultimately cause cell membrane disruption, thereby activating diverse cell death mechanisms including apoptosis, necrosis, and ICD [52, 61, 62, 63]. PIT is clinically approved for the treatment of recurrent EGFR‐expressing head and neck cancer patients and has demonstrated substantial antitumor immune activities when combined with immune checkpoint blockade therapy (NCT04305795) [63, 64, 65]. Therefore, this review aims to provide an overview of the current clinically approved TNBC therapies, present their limitations, and discuss the therapeutic potential of light‐based PIT as an effective and nontoxic immunotherapeutic option.

### 1.2 Non‐Targeted Breast Cancer Therapy

#### 1.2.1 Chemotherapy and Breast Cancer Treatment

Systemic chemotherapy (oral or intravenous) remains the foundation for preventing recurrence in stage I–III breast cancer [66, 67]. Neoadjuvant chemotherapy (NAC), initially developed for buccal carcinoma in the 1980s [68, 69], was adopted in breast cancer to reduce tumor burden prior to surgery or radiotherapy (RT) [70]. As evidence demonstrated equivalent survival outcomes between breast‐conserving therapy and mastectomy, NAC use was expanded to facilitate organ preservation in locally advanced disease [71, 72, 73]. Adjuvant chemotherapy (AC) targets micrometastases present at surgery, improving long‐term survival and reducing recurrence [74, 75, 76]. Current evidence suggests neither AC nor NAC demonstrates superior survival benefits across breast cancer subtypes [77, 78], with comparable outcomes in disease progression, disease‐free survival, and overall survival regardless of treatment sequence [77, 78, 79, 80, 81].

The foundation of modern breast cancer chemotherapy was established in the 1970s with the identification of several cytotoxic drug classes: alkylating agents (cyclophosphamides, thiotepa), antimetabolites (5‐fluorouracil, methotrexate), vinca alkaloids (vincristine), and anthracyclines (doxorubicin) [82, 83]. Current standard regimens for early‐stage disease combine anthracyclines and taxanes over 18–24 weeks [84]. However, widespread adjuvant use of these agents has led to increased pre‐treatment in metastatic cases, complicating subsequent management [76]. For TNBC specifically, platinum‐based combinations with anthracycline‐taxane therapy are now recommended to enhance pathological complete response rates [84, 85, 86].

Anthracyclines remain among the most effective chemotherapeutic agents for breast cancer, with multiple trials demonstrating superior recurrence‐free and overall survival compared to CMF (cyclophosphamide, methotrexate, and fluorouracil) regimens [87]. By 2000, 70%–80% of women under 70 received adjuvant anthracyclines [88]. However, their clinical use has declined due to dose‐dependent cardiotoxicity, which can lead to irreversible congestive heart failure [89, 90]. This safety concern has driven investigation into taxane‐based alternatives. Taxanes (paclitaxel, docetaxel) exert antitumor effects by stabilizing microtubules and blocking mitosis [76]. While paclitaxel showed significant activity in metastatic breast cancer during the 1990s, docetaxel demonstrates superior pharmacokinetics with a longer half‐life and enhanced cellular retention [76, 91]. A key limitation remains intrinsic and acquired resistance, primarily mediated by p‐glycoprotein overexpression [92].

#### 1.2.2 Surgery

As the oldest oncological discipline with roots tracing to ancient egypt [93], surgical intervention remains a cornerstone of cancer treatment. Modern surgical approaches have evolved significantly with the advent of robotics, laparoscopy, and advanced reconstructive techniques, all aimed at reducing morbidity while preserving form, function, and quality of life [94]. Contemporary oncologic surgery emphasizes precise tumor excision with adequate margins of healthy tissue, rather than direct tumor manipulation [94]. This precision‐based approach requires sophisticated imaging guidance, currently provided by X‐rays, RT, and CT scans to accurately delineate tumor boundaries [95]. The ongoing surgical challenge lies in balancing complete local control with optimal cosmetic outcomes and minimal recurrence risk [94]. Recent progress in surgical techniques, combined with the widespread adoption of AC/NAC, has created new opportunities for breast conservation and reconstruction following mastectomy [77].

In the 1980s, mastectomy with axillary lymph node dissection was standard treatment, often leaving women with permanent disfigurement that necessitated prosthetic use and significantly impacted body image, sexuality, and feminity [94]. Surgical disfigurement is well documented to cause psychological distress, increasing vulnerability to depression [96, 97]. Common physical sequelae include lymphedema, reduced shoulder mobility, and diminished upper extremity strength [98, 99]. Notably, some patients with unilateral disease elect for bilateral mastectomy despite lacking evidence for improved survival [100, 101]. Treatment decisions between lumpectomy and mastectomy are influenced by multiple factors: tumor characteristics (size, location), treatment access (radiotherapy availability), and patient demographics (socioeconomic status, education, ethnicity, BRCA status, and family history) [102, 103, 104, 105, 106, 107].

The field of postmastectomy reconstruction has made significant progress, with innovative plastic surgery techniques leading to steadily increasing reconstruction rates. Multiple studies demonstrate that reconstruction improves quality of life and mental health outcomes for mastectomy patients [108, 109], though these women may experience greater physical discomfort compared to other surgical options [110]. Breast‐conserving surgery, pioneered by Fisher and Veronesi [71, 111] has emerged as a safe alternative to mastectomy. Early conservation approaches involved radical quadrantectomy without contour restoration [94], but modern oncoplastic techniques now allow excision of up to 50% of breast volume while preserving esthetics through sophisticated intramammary flaps and pedicle techniques [112].

These advancements have ushered in a renaissance for cancer surgery, particularly with the emergence of personalized surgical approaches. Cutting‐edge techniques utilizing intraoperative molecular imaging, including specialized dyes and radioactive isotopes, are rapidly developing [113]. However, questions remain regarding the cost‐effectiveness and practical implementation of these innovations in standard clinical practice.

#### 1.2.3 Radiotherapy

The foundations of breast cancer radiation therapy were established in the 1930s following the pioneering work of Röntgen and Curie, utilizing radium needles and orthovoltage X‐rays [114, 115]. Initially employed either adjuvantly after mastectomy or as primary treatment for inoperable cases [116], RT has since become integral to breast conservation. Modern protocols combining surgery with radiation reduce 10‐year recurrence risk by 50% and lower 15‐year breast cancer mortality by 20% [117, 118, 119], while significantly improving bilateral preservation rates [120]. However, radiation's benefits are context‐dependent: while it reduces local recurrence across age groups, survival improvement is not observed in older patients (> 70 years) receiving hormonal therapy [121]. Surprisingly, approximately 15% of younger women with early‐stage disease forego RT despite its established benefits [122, 123]. Common barriers include: (1) suboptimal patient selection; (2) subsequent mastectomy after initial breast conservation; (3) logistical challenges (transportation/access) and concern about treatment toxicity [124].

While postmastectomy radiation therapy (PMRT) remains standard for advanced breast cancer (tumors > 5 cm or > 4 involved lymph nodes) [87, 125, 126, 127], its expanding use stems primarily from improved local‐regional control rather than survival benefits or prevention of distant metastases [116]. Significant limitations persist, including targeting challenges such as imaging that cannot reliably identify all regional disease or delineate optimal volumes [116]. Other challenges include toxicity concerns related to non‐specific radiation exposure risks, capable of causing RT‐induced dermatitis, pulmonary fibrosis, cardiac complications and secondary malignancies [128, 129, 130, 131, 132]. Recent advances in 3D CT0‐based planning and precision delivery techniques aim to enhance tumor targeting while minimizing normal tissue exposure and associated toxicities [133].

Accelerated partial‐breast irradiation (APBI) delivers higher RT doses over a few days while maintaining comparable efficacy to conventional therapy [134]. Three primary delivery methods exist: (1) external‐beam radiation, providing excellent target coverage with lower conformality than brachytherapy, potentially increasing exposure to adjacent healthy tissues [135, 136]. Advanced techniques like intensity‐modulated RT and CT‐guided tomotherapy may mitigate these limitations [137]. (2) Brachytherapy uses implanted catheters to minimize normal tissue exposure, though the infection risk remains [135]. (3) Intraoperative radiation delivers a single high‐dose treatment during surgery, offering logistical and psychological benefits [136, 138]. This approach capitalizes on the finding that 85% of recurrences occur in the original tumor quadrant [139]. While initial safety profiles appear favorable, long‐term cosmetic outcomes require further evaluation due to potential radiation‐induced fibrosis [138].

#### 1.2.4 PDT

PDT represents a minimally invasive treatment approach for both malignant and benign conditions [140]. The therapy utilizes PS that, when activated by specific light wavelengths, generate ROS (singlet oxygen, radicals) that induce oxidative damage to cellular components (DNA, lipids, proteins) [141, 142, 143, 144]. This oxidative stress disrupts critical signaling pathways and gene regulation, ultimately leading to cell death. Recent evidence highlights PDT's unique dual capability: direct tumor destruction combined with immune activation against metastases [145, 146]. Compared to conventional therapies, PDT offers reduced morbidity while addressing both local and distant disease.

**FIGURE 1:**
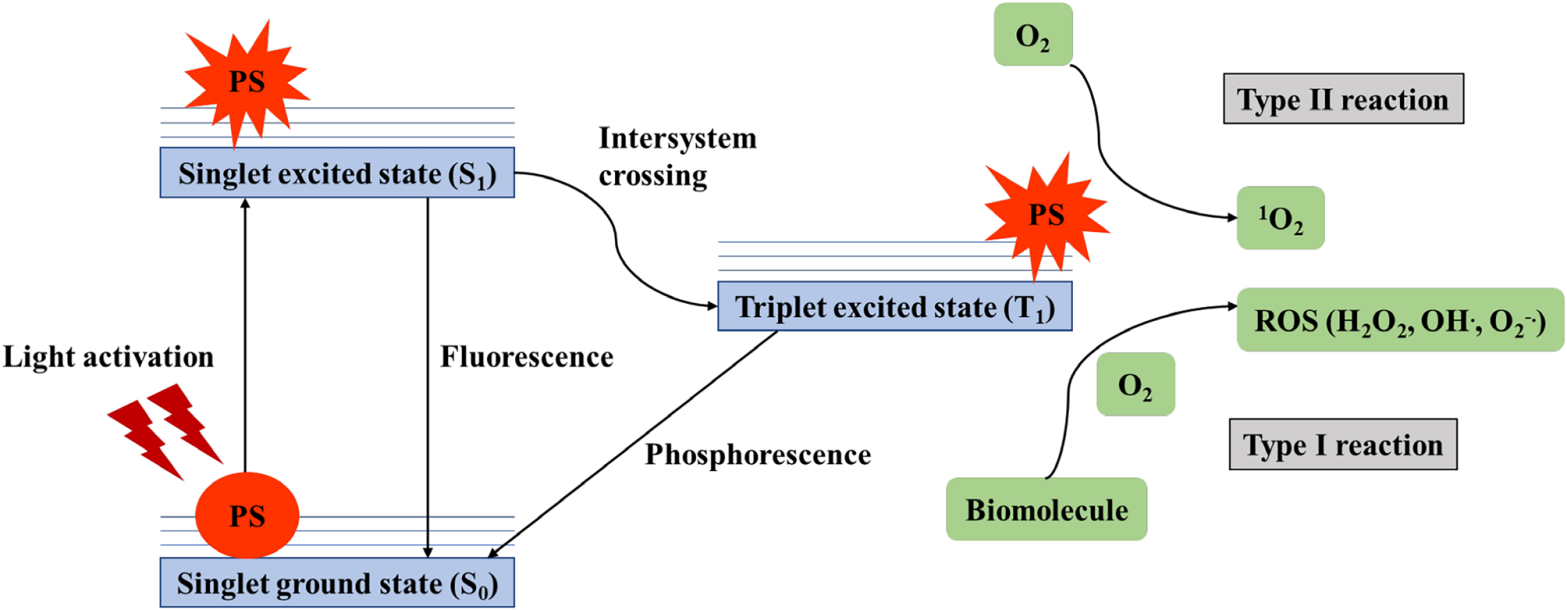
Principle of photodynamic therapy. Following light absorption, the photosensitizer reaches an excited singlet state (S_1_). Transition to the triplet excited state (T_1_) allows two primary pathways to become feasible: (1) Type‐I reaction, whereby the photosensitizer reacts with biomolecules or directly transfers electrons/hydrogen atoms to generate radicals (e.g., superoxide anion, hydroxyl radical), which in turn react with molecular oxygen to generate reactive oxygen species, or (2) Type‐II reaction, where energy is transferred directly to oxygen to form singlet oxygen species (^1^O_2_). Both pathways foster the therapeutic efficacy of PDT via oxidative damage of target tissues.

PDT requires three nontoxic components acting in concert: (1) a PS, (2) tissue oxygen, and (3) visible/near‐infrared (NIR) light (600–800 nm wavelength) [140]. Effective treatment depends on achieving optimal PS concentration in target tissue prior to light activation [146]. The photochemical process initiates when the PS absorbs light (600–800 nm), promoting its transition from ground state (*S*_0_) to an excited state (S_1_) (Figure 1) [145, 146, 147, 148]. This wavelength range is particularly effective for therapeutic applications because it minimizes absorption by endogenous chromophores (hemoglobin, water, and proteins), thereby enhancing light penetration to deeper tissues. Through intersystem crossing from singlet (S_1_) to triplet (T_1_) state, the excited PS transfers energy to either: (1) biomolecules, generating ROS via a type‐I mechanism, or (2) molecular oxygen, producing cytotoxic singlet oxygen (^1^O_2_) via a type‐II mechanism (Figure 1) [146, 149].

The type‐II reaction (singlet oxygen generation) is considered the primary driver of PDT efficacy [149]. However, the relative contribution of type‐I (ROS‐mediated) and type‐II mechanisms depends on multiple factors, including oxygen tension, tissue microenvironment (pH, dielectric constant), and PS properties [149]. Notably, oxygen depletion shifts the balance toward type‐I reactions [150]. PDT induces tumor destruction through multiple synergistic cell death pathways determined by the subcellular localization. For instance, apoptosis is induced following mitochondrial localization, and necrosis through membrane targeting, whereas autophagy is triggered post‐lysosomal and endoplasmic reticulum (ER) accumulation [151, 152, 153]. Additionally, vascular damage disrupts tumor perfusion while stimulating antitumor immunity through inflammatory activation [149, 154, 155]. This multimodal cell death induction represents a key therapeutic advantage, overcoming resistance to individual death pathways [156].

The selective tumor destruction in PDT stems from the preferential PS accumulation in malignant tissue, enabled by: (1) enhanced permeability and retention effect (EPR) from leaky tumor vasculature; (2) increased LDL receptor‐mediated uptake in proliferating cells; and (3) impaired lymphatic drainage [146, 149, 157]. Currently, few clinically approved PS exist due to stringent photochemical requirements [145]. Key agents for breast cancer include: first‐generation‐PS such as porfimer sodium (Photofrin), which is FDA‐approved for multiple cancers but limited by chemical heterogeneity, prolonged skin photosensitivity, and suboptimal tumor penetration [146, 148, 158, 159, 160, 161, 162, 163, 164]. Other agents are verteporfin, temoporfin, and lutetium texaphyrin, developed to overcome these limitations with improved purity and pharmacokinetics [145, 158, 162, 163, 164, 165]. The 1993 approval of Photofrin marked PDT's clinical translation, though its drawbacks drove the development of superior second‐generation compounds [162, 163, 164].

Following its approval for head and neck cancers [166, 167], temoporfin (mTHPC) demonstrated variable efficacy across malignancies, though showed particular promise for breast cancer with its minimally invasive profile and favorable safety [168, 169, 170]. Among other clinically relevant PSs, visudyne has shown to be effective against cutaneous breast cancer metastases despite hydrophobicity, offering beneficial properties such as optimal tissue penetration at 690 nm; wider therapeutic window of 30–150 min and complete systemic clearance within 48 h [160, 163, 169]. Despite its poor water solubility, Visudyne exhibits high tissue penetration at 690 nm, with an optimum. Other agents such as purlytin, talaporfin, and lutetium texaphyrin have shown activity against metastatic disease, recurrence, and skin lesions [162, 163, 164]. Current research explores synergistic approaches combining PDT with conventional adjuvant therapies (chemotherapy and RT) to enhance treatment outcomes.

PDT demonstrates synergistic effects with conventional therapies such as RT, as demonstrated by the enhanced tumor cell killing and reduced adverse effects observed when combining indocyanine green‐mediated PDT with low RT doses [159, 171]. However, PDT combined with chemotherapy such as doxorubicin has shown to enhance antitumor activity in a breast cancer model [154, 172, 173]. Similarly, synergy was observed with dacarbazine (DTIC) and hypericin‐PDT combination (HYP‐PDT) in treating chemo‐resistant melanoma [174]. Of note, HYP‐PDT has been reported to trigger ICD through the release of danger‐associated molecular patterns (DAMP: e.g., CRT, ATP, HSP70/90) immunological signals, capable of recruiting and activating innate immune cells (macrophages, dendritic cells), which subsequently promote tumor antigen presentation in secondary lymphoid organs (e.g., spleen and lymph nodes) to activate cytotoxic CD8+ T‐cells against both locally irradiated and distant non‐irradiated tumors [56, 59, 175, 176, 177]. However, multiple studies have reported the dependence of PDT‐induced antitumor immune response on tumor vasculature integrity [178, 179]. For instance, Henderson et al., 2004 showed how using high‐dose PDT (128 J/Cm^2^) could cause complete vascular destruction, impairing immune cell infiltration [180]. Conversely, Shams et al., 2015 demonstrated how low‐dose PDT (48 J/cm2) induces vascular leakiness, facilitating the infiltration of antitumor immune cells within irradiated tumors [181]. Therefore, optimal therapeutic outcomes require combination approaches to address both primary and metastatic lesions [181].

While PDT demonstrates advantages over conventional therapies, including improved quality of life and reduced treatment costs, several barriers limit its clinical application [182, 183]. These barriers comprise [1] oxygen depletion in targeted tissues, [2] limited light penetration depth, [3] off‐target effects (photosensitivity, pain), and [4] incomplete tumor eradication and treatment resistance [145, 146, 154, 184]. These challenges currently preclude PDT from being a first‐line option for most malignancies, including breast cancer [184]. Therefore, the emergence of ADCs offers potential solutions by improving targeted drug delivery and therapeutic index [185].

### 1.3 Targeted Cancer Treatment

#### 1.3.1 Antibody Drug Conjugates

**FIGURE 2:**
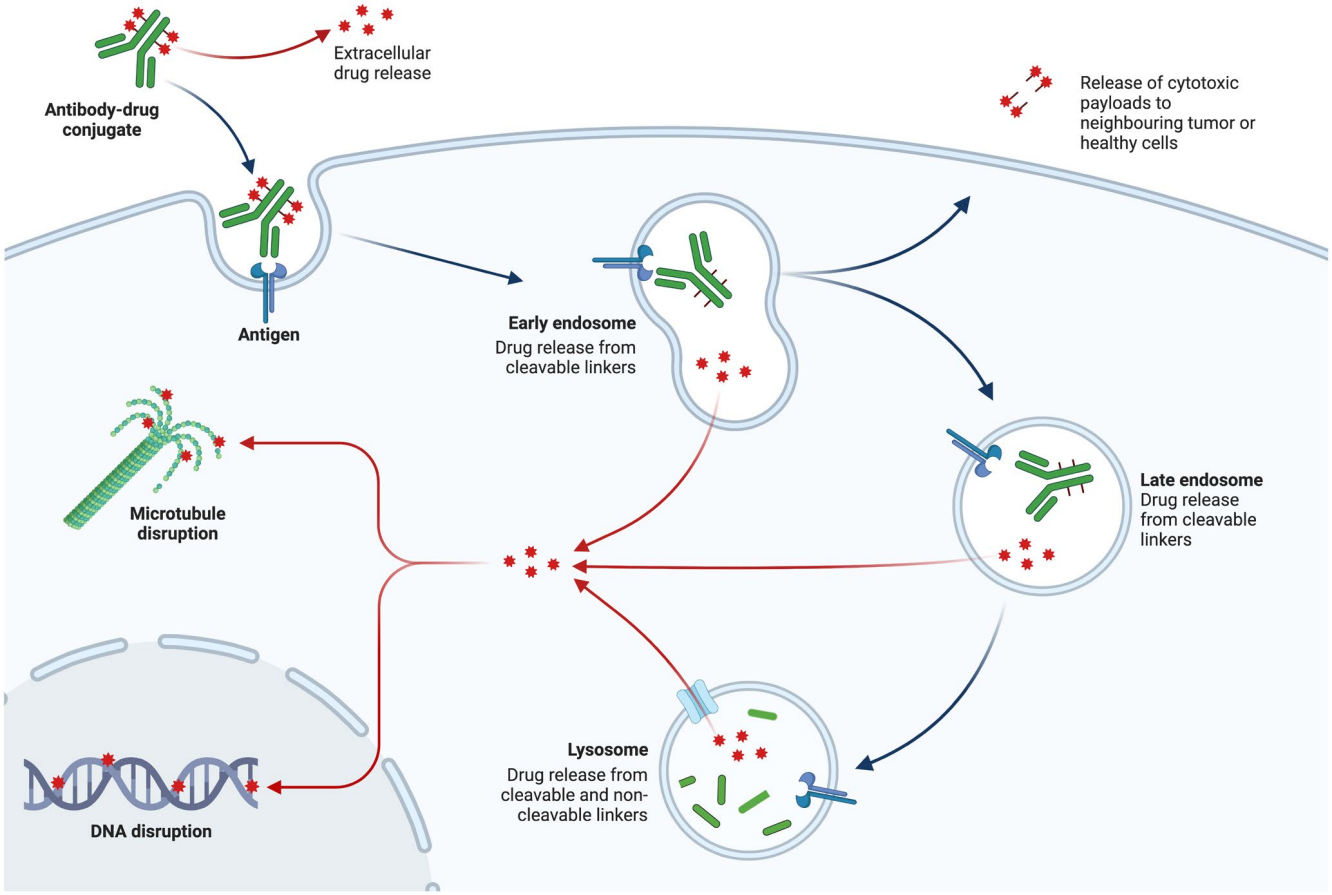
Mechanism of action of antibody drug conjugated to anti‐mitotic agents. The ADC binds specifically to its target antigen on the surface of the tumor cell, eliciting internalization via endocytosis. Once inside the cell, the linker is cleaved in the lysosomal compartment, releasing the anti‐mitotic payload, which in turn binds to microtubules and inhibits tubulin polymerization. This phenomenon results in cell cycle arrest, typically at the G_2_/M phase, culminating in the apoptosis of target cells.

ADCs represent a breakthrough in targeted cancer therapy, combining mAbs with potent cytotoxic payloads via specialized linkers [186, 187]. This design capitalizes on tumor antigen specificity to deliver cytotoxic drugs directly to cancer cells while minimizing systemic toxicity (Figure 2) [188]. Despite early setbacks due to pharmacological challenges [186, 189, 190], advances in three key components, including (1) antibody engineering (improving targeting); (2) linker technology (enhanced stability), and (3) payload optimizations (increased potency) have revitalized ADC development [191, 192, 193]. After three decades of research, only 11 ADCs, such as Gemtuzumab ozogamicin (Mylotarg), Brentuximab vedotin (Adcetris), Trastuzumab emtansine (Kadcyla/T‐DM1), Inotuzumab ozogamicin (Besponsa), Polatuzumab vedotin‐piiq (Polivy), Enfortumab vedotin (Padcev), Trastuzumab deruxtecan (Enhertu), Moxetumomab pasudotox (Lumoxiti), Sacituzumab govitecan (Trodelvy), Belantamab mafodotin‐blmf (Blenrep), and Loncastuximab tesirine‐lpyl (Zynlonta), have gained clinical approval, including two targeted agents for metastatic breast cancer (Trastuzumab emtansine (2013) and Trastuzumab deruxtecan (2019)) [194, 195]. More recently, Sacituzumab govitecan (anti‐TROP‐2) received FDA approval (2021) for advanced TNBC [53], marking a significant advancement in ADC therapeutics (Figure 2).

##### 1.3.1.1 The Linker Types and Antibody Drug Conjugate

The overall biophysical and physiological properties of ADCs are determined by their three structural components, namely the antibody, the linker, and the cytotoxic warhead, as well as the bioconjugation method used to assemble them [186, 188]. These elements are critical in designing effective ADCs.

The monoclonal antibody (mAb) component must be carefully selected based on target antigens that meet specific criteria: (1) high expression on tumor cells with minimal presence in healthy tissues, (2) surface localization for accessibility to circulating mAbs, and (3) efficient internalization to facilitate ADC uptake and payload delivery. Additionally, the mAb should exhibit high specificity, strong binding affinity, prolonged retention, low immunogenicity, and minimal cross‐reactivity [196, 197, 198, 199, 200, 201].

The linker, which connects the antibody to the cytotoxic payload, plays a crucial role in ADC stability and efficacy. It should remain intact in circulation to prevent off‐target drug release while enabling efficient payload liberation after internalization and trafficking to specific subcellular compartments [202, 203, 204]. Linkers fall into two broad categories: cleavable and non‐cleavable. Noncleavable linkers (e.g., thioether‐based) require complete antibody degradation in lysosomes to release the drug, as seen in trastuzumab emtansine (T‐DM1) [205]. In contrast, cleavable linkers are designed to break under specific conditions (e.g., pH, redox, potential, or enzymatic activity), allowing controlled payload release [190]. Trastuzumab deruxtecan (anti‐HER2 mAb conjugated to topoisomerase I inhibitor Dxd, a derivative of camptothecin analogue exatecan), for instance, employs a tetrapeptide‐based cleavable linker for enhanced systemic stability [192, 205, 206, 207]. The cytotoxic payload must also meet stringent criteria, including sub‐nanomolar potency, aqueous solubility, low molecular weight, extended half‐life, and minimal immunogenicity. Moreover, it should conjugate effectively to the linker without compromising antibody internalization while enhancing antitumor activity [208, 209, 210].

ADC warheads primarily fall into two categories: microtubule‐disrupting agents (e.g., auristatins and maytansinoids) and DNA‐damaging agents (e.g., calicheamicin, duocarmycin, and doxorubicin) [201]. For example, trastuzumab emtansine (T‐DM1) delivers DM1, a maytansinoid derivative that inhibits tubulin polymerization, arresting the cell cycle at the G2/M phase and inducing apoptosis. In contrast, trastuzumab deruxtecan (T‐DXd) employs a topoisomerase I inhibitor (DXd), which disrupts DNA replication, leading to cell cycle arrest and programmed cell death [201]. T‐DM1 gained FDA approval following two phase‐III trials demonstrating improved progression‐free and overall survival in metastatic breast cancer patients. With an average drug‐to‐antibody ratio (DAR) of 3.5, it ensures selective tumor targeting while minimizing off‐toxicity [195, 206, 207, 211, 212]. Meanwhile, T‐DXd, with a higher DAR (7.7), enhances payload delivery per antigen engagement and exhibits a bystander effect, killing nearby HER2‐negative cells—a feature absent in T‐DM1 [213]. However, this effect can also lead to unintended cytotoxicity, as seen in trials with bivatuzumab mertansine, where mertansine accumulation in healthy skin cells caused fatal epidermal necrolysis [214].

Interestingly, Robert et al. have reported the reduced in vivo potency, rapid hepatic uptake, and plasma clearance of anti‐CD70/CD30 mAbs conjugated to monomethyl auristatin‐F (MMAF) using the protease‐cleavable valine citrulline‐*p*‐aminobenzylcarbamate (val‐cit‐PABC) linker [215, 216]. These results were contrasting with their in vitro data, showing enhanced potency with increasing DAR ratios [215, 216]. This dichotomic behavior of MMAF‐based ADCs was attributed to the added hydrophobicity of the val‐cit‐PABC linker, which exhibited longer retention on hydrophobic interaction chromatography (HIC) compared to their ADC counterparts deprived of the abovementioned linker [215]. The implication of the linker hydrophobicity and the dependence on drug loading ratio was further validated by the superior antitumor activity of ADCs with DAR 4 over those with DAR 8 ratio [215, 216]. This observed therapeutic discrepancy was confirmed when opposite effects were obtained when substituting val‐cit‐PABC with hydrophilic linkers [215, 216]. As expected, these hydrophilic linker‐based ADCs displayed substantially improved antitumor and pharmacokinetic activities, positively correlating with an increased DAR ratio of 4 to 8 [215, 216]. Of late, SGN‐CD228A, a melanotransferrin mAb conjugated to 8 MMAE molecules (DAR of 8) using glucuronide hydrophilic linker instead of a valine citrulline, recently progressed to a phase‐I clinical trial for the treatment of melanoma, mesothelioma, and TNBC patients expressing melanotransferrin receptor (NCT04042480) [217]. However, the later trial was interrupted supposedly due to management prioritization. Altogether, these adverse events depicted in ADC trials are not related to the normal tissue expression of targeted TAAs but rather due to the types of linkers and cytotoxic payloads used [218]. Given that most drugs rely on hydrophobic moieties to exert their potency, it becomes of paramount importance to develop alternative linker‐payload chemistries with stealth properties to generate more effective ADCs. The most common method for masking the linker hydrophobicity is PEGylation, a process whereby a polyethylene glycol (PEG) polymer is inserted as a spacer between the drug payload and antibody point of attachment [215].

##### 1.3.1.2 Minimizing ADCs Related Antigen Independent Toxicity and Non‐Specific Rapid Clearance Through PEGylation

Since PEG was first introduced as a cloaking technique, it has become common practice to covalently couple PEG molecules to therapeutic proteins to improve their pharmacokinetic properties [219, 220, 221]. PEGs are commonly used, FDA‐approved, biocompatible, nontoxic, synthetic polymers that have a plethora of uses in various industries, including the biomedical sciences, food sciences, and cosmetics, among others. Its molecular structure includes flexible polymers in a chain formation, which can be linear or branched, typically with a molecular weight between 0.4 and 40 kDa. The chain links comprise 10 oxyethylene glycol subunits each, arranged in S‐shaped fragments. This structure creates cavities between links, which reportedly allow for the adsorption of 2 to 3 molecules of H_2_O; this accounts for PEG's hydrophilicity, its pharmacokinetic behavior that follows that of a much larger molecule, and its ability to cloak biopharmaceutical structures from their environment by increasing their water solubility [222, 223, 224]. PEG, together with its associated H_2_O molecules, forms a protective shield that can prevent enzymatic degradation and proteolysis, inhibit interactions with cell surface proteins, and slow renal clearance; overall, adverse immunological effects are limited by adding PEG. Moreover, PEGylation increases stability over a range of pH levels and temperatures [225]. PEG shows limited in vivo toxicity, and studies show its intact elimination from the body via the kidneys for molecules less than 30 kDa or the feces for molecules larger than 20 kDa [226]. PEGs can be synthesized with different terminal groups, allowing for attachment to various molecules [224], and by conjugating PEG to therapeutic proteins, such as antibodies, or other therapeutic components, such as nanocarriers, we can confer these abovementioned properties to the nanobiopharmaceutical. This increases their stability and hydrophilicity and consequently decreases their tendency to aggregate while improving upon several pharmacokinetic properties, including serum half‐life, biodistribution, drug availability, and clearance [227, 228, 229]. Additional benefits include reduced immunogenicity (i.e., the ability to induce an immune response), reduced antigenicity (recognizability by antibodies generated due to an immunogenic response), reduced toxicity, masking of non‐specific recognition sites, and resistance to proteolytic degradation and anti‐drug antibodies (due to reduced accessibility) [230, 231]. In developing PEGylated proteins, researchers must consider that the observed effects of PEGylation are correlated with the PEG molecules' backbone structure and complexity (i.e., whether chains are linear or branched) and molecular weight. Even minuscule changes to PEG size, in particular, can drastically alter a protein's pharmacokinetics [231, 232]. Nonetheless, while PEGylation provides a plethora of invaluable advantages, these should not be sought at any cost. It is essential that during the PEGylating process, the antibody–antigen binding affinity is preserved, as well as the Fc functionality if required. Thus, chemistry must be carefully considered. Davis et al. [233], were the first to develop the PEGylation chemistry and report the endowment of proteins with a protective shield.

In general, to form a stable bond between a PEG polymer and a protein, PEG must first be chemically activated so it is able to react with the protein [223]. Several chemistries have been developed to prepare active PEG derivatives with various functional groups that enable coupling to proteins; these include, but are not limited to, active carbonate, amine, ester, aldehyde, or tresylate groups [223]. The resulting activated PEG molecule can then be covalently linked to a reactive site on the protein, most commonly via the α or ε amino groups of a lysine residue, using amine‐reactive chemistry 231. Here, PEG molecules are typically functionalised with an N‐hydroxysuccinimide ester, facilitating the formation of an amine bond between the PEG molecule and the antibody residue. Due to the number of available lysine residues on a single antibody, first‐generation PEGylation methods using the lysine amino groups typically result in the uncontrollable modification of multiple lysine residues, producing heterogeneous mixtures of PEG isomers with varying molecular masses because this method allows for PEG to be attached in various locations throughout the protein [234]. Thus, each antibody has a varying degree of PEGylation; PEG is not added in a consistent ratio [234]. Consequently, drug batches are difficult to reproduce using this chemistry, and the antigenicity of the drug can be affected, as well as the clinical outcomes of such heterogeneous formulations [223]. Second‐generation chemistry aimed to alleviate these problems associated with first‐generation methods, and an array of methods now exist [235]. For example, further research has considered the use of cysteine residues as the PEGylation sites because these are significantly less abundant on proteins than are lysine groups. Cysteine thiol groups are, therefore, ideal for achieving more specific modifications. Furthermore, using genetic engineering, cysteine can be added precisely to selected regions of the protein. Site‐specific PEGylation can minimize immunogenicity and curtail the loss of biological activity [236, 237, 238]. Second‐generation PEGylation methods have also explored the incorporation of branched PEG structures [225], which behave pharmacokinetically as if they are larger than linear PEG molecules of the same molecular mass [239]. Branched structures provide more effective stealthing and, therefore, cloak the protein more effectively from the host immune system and proteolytic enzymes, resulting in lower antigenicity and reduced protein destruction [240]. Nonetheless, second‐generation PEGylation has been shown to reduce the bioactivity of the antibodies. Thus, third‐generation PEGylation methods, which aim to retain bioactivity without sacrificing pharmacokinetic benefits, are currently under development [241, 242]. Complete descriptions of all PEGylation chemistries are beyond the scope of this review; however, comprehensive reviews of the common techniques developed since Davis et al.'s work in the 1970s are available in the literature [235, 243]. To date, a number of PEGylated proteins have been approved for clinical use, while others are undergoing trials [220, 244].

##### 1.3.1.3 Enhancing Pharmacokinetic Behavior of ADCs

Our improved understanding of the limitations of mAbs, coupled with advances in recombinant DNA technologies, has led to the development of protein‐engineered antibody fragments of different formats, including reduced sizes, providing various associated advantages, such as increased tissue penetrability and uptake. Nonetheless, when the size becomes too small, certain disadvantages come into play, particularly relating to rapid systemic clearance and decreased serum half‐life, as well as reduced stability and antigen affinity compared to full‐length mAbs [245, 246]. One of the advantages of decorating antibodies or their fragments with PEG is that this increases the hydrodynamic radius of the protein. By increasing the size of the structure, especially if increased above the threshold for glomerular filtration, a chain reaction of important pharmacokinetic changes occurs: first, the rate at which the antibody is cleared from systemic circulation (due to size, proteolysis, and immune [223]) is decreased; second, this inherently means that the serum half‐life is increased; third, the bioavailability of and thus exposure to the biopharmaceutical is increased; fourth, uptake of the biopharmaceutical by cancer cells increases; and finally, the efficacy of the therapy is enhanced while the dosage and dose frequency can be reduced, making it easier to optimize the therapeutic index [223, 225, 236, 247, 248]. This effect has been demonstrated in various antibody fragment formats.

In 2018, Li et al. investigated how PEGylation affected the in vivo pharmacokinetics and tumor uptake of an anti‐oncofetal antigen 5T4 diabody [249]. They demonstrated that the size of the attached PEG was correlated with the conjugate clearance rate, stating that diabodies decorated with larger PEGs exhibited slower systemic clearance. In particular, their findings indicated that 6 nm was the hydrodynamic radius threshold for glomerular clearance of their structure. The reduced clearance rates had a direct effect on the serum half‐life of the PEGylated vs. unPEGylated diabodies. The unPEGylated diabody (with a hydrodynamic radius of 2.9 nm) had a half‐life of 40 min. Conversely, diabodies conjugated with PEG with a molecular weight of 5 kDa (giving them a hydrodynamic radius of 3.0–3.3 nm) had a half‐life of 4.0 h for linear PEG structures and 4.8 h for branched PEG structures. When branched PEG of 40 kDa was utilized (increasing the hydrodynamic radius to 12 nm), the half‐life increased dramatically to over 43 h. Moreover, the blood concentration increased 10‐fold for linear 5 kDa PEG, 20‐fold for branched 5 kDa PEG, and 263‐fold for branched 40 kDa PEG, meaning that the bioavailability of the diabody was increased significantly through PEGylation, proportionally to PEG size and complexity. Their results further showed that the in vivo biodistribution and, ultimately, the tumor uptake of their modified diabody was significantly associated with PEG size and shape (linear or branched). They suggested that hydrodynamic size was a better predictor of pharmacokinetic behavior than molecular weight for evaluating PEGylated biomolecules and also noted that other biophysical properties, such as surface charge, might also play a role in clearance and half‐life.

In 2018, Pan et al. [250] similarly studied thiol‐specific PEGylation of bispecific antibody fragments targeting the carcinoembryonic antigen (CEA) and CD3 for human colon adenocarcinoma (PEG‐S‐Fab) and reported increased half‐life, plasma stability, and in vivo cytotoxic effect in mice. Their PEG‐S‐Fab construct exhibited a 12‐fold increase in serum half‐life compared to its unPEGylated counterpart (S‐Fab) and a more potent antitumoral effect in a xenograft mouse model. They showed that PEG‐S‐Fab retained its specificity for both CEA and CD3, with minimal reduction in affinity. In human plasma, enzymatic digestion led to rapid declines in S‐Fab, but the stability of PEG‐S‐Fab showed slower decreases. Finally, while in vitro cytotoxicity was slightly lower for PEG‐S‐Fab (likely because the decreased binding affinity indicated steric hindrance by PEGylation occurring near the antigen binding site), the in vivo cytotoxicity was significantly higher, likely due to the increased half‐life and stability, which was able to overcome the effects on binding affinity by increasing drug exposure. Similar findings on the in vitro against in vivo bioactivity of PEGylated proteins have also been demonstrated elsewhere [251]. Based on their findings, Pan et al. have recommended that PEGylated S‐Fab bispecific antibody fragments offer a more feasible biopharmaceutical agent for further study. The use of PEGylation to oligomerize GD2‐specific scFv fragments was reported by Kholodenko et al. [245] in 2019. Their study showed that PEG‐based multimerization of the scFv monomers resulted in improved blood‐to‐background and tumor‐to‐background ratios, slower decreases in protein concentration, reduced cell viability, and increased DNA fragmentation, with no cross‐reactivity between fragment monomers.

##### 1.3.1.4 Caveat: Anti‐PEG Immunogenicity

It must, however, be noted that despite the wide use of PEGylated therapeutic proteins and previous assertions that PEG is a weak immunogen [52], research has recently indicated that there are potential risks associated with the development of anti‐PEG antibodies and subsequent immune response [252]. It has been suggested that this limits how many doses of PEGylated drugs an individual has received before an anti‐PEG immunogenic reaction accelerates systemic clearance and renders the drug ineffective [253, 254]. Some studies have even observed anti‐PEG antibody production in healthy individuals with no history of PEGylated drugs, potentially due to the increasing presence of PEG in food items, cosmetics, and other household items [255, 256, 257]. Our increasing exposure to PEG over the years has led to more and more of the population having pre‐existing anti‐PEG antibodies prior to any exposure to PEGylated drugs [258, 259]. However, some research has shown that determining the ideal dosing interval can decrease anti‐PEG antibody production and limit accelerated blood clearance [254, 260]. Furthermore, it is suggested that the innate immunogenicity of the protein itself can influence immunogenic responses to PEG, and the PEG molecular weight and grafting density also strongly influence host responses [253]. Taken together, it appears that while biopharmaceutical developers must approach PEGylation with care, taking the right precautions makes it possible to strike a balance, harnessing the beneficial pharmacokinetic advantages of PEG while mitigating the immunogenic obstacles.

Nonetheless, with these recent concerns regarding the immunogenicity of PEG, researchers have been exploring alternative stealthing methods [261], one of which is the use of branched PEG structures, which are reportedly less antigenic due to their more complex structure [262]. Another option is the use of poly(2‐oxazoline), which is a hydrophilic, nontoxic polymer similar to PEG that is synthesized from 2‐oxazoline monomers [263]. It offers high stability, easy hetero‐functionalization, and high biocompatibility. This relatively new arrival is being studied for its use in therapeutic polymers [263]. Of interest are also Poly(zwitterions), which are polyelectrolytes made up of monomers comprising zwitterionic moieties that possess groups with both positive and negative charges but are overall neutral [264]. They provide highly hydrophilic surfaces, which are able to decrease the adsorption of proteins onto a nanoparticle (NP) surface, enabling them to improve serum half‐life and escape the immune surveillance [265]. NPs have even undergone cell membrane coating, whereby natural cell membranes are isolated and physically cloaked onto the NP surface to confer cell‐like properties to the NP, allowing them to bypass immune recognition [266] While PEGylation remains the gold standard for stealthing biopharmaceutical products, research is ongoing into these and other techniques to provide a site‐specific drug conjugation method with reproducible stoichiometry, enabling the production of homogeneous immunoconjugates with less variable pharmacokinetic activities.

#### 1.3.2 The Potential of Site‐Specific Conjugation Methods for ADCs

Current ADC development primarily relies on two conventional conjugation methods: lysine side‐chain functionalization and disulfide bond reduction to generate reactive sulfhydryl groups [267]. However, these approaches produce heterogeneous ADC mixtures with drug‐to‐antibody ratios (DAR) ranging from 0 to 8, leading to variable pharmacokinetic and toxicity profiles [268]. This heterogeneity stems from the numerous available conjugation sites. Antibodies contain multiple cysteine residues (after reduction) and dozens of lysine side chains, potentially creating millions of conjugation variants [267, 269]. As highlighted by Hamblett et al. (2004), the number of conjugated drug molecules per antibody is a critical determinant of ADC efficacy [216]. This understanding has driven the development of site‐specific conjugation strategies enabled by advances in genetic engineering. Modern techniques now allow the production of recombinant antibodies with precisely engineered modifications for controlled, uniform drug attachment, addressing the limitations of traditional conjugation methods [270, 271, 272].

##### 1.3.2.1 Sortase‐Mediated Enzyme Conjugation

Sortase A, a transpeptidase from *Staphylococcus aureus*, catalyzes site‐specific conjugation by forming an amide bond between the C‐terminal threonine of an LPXTG motif and the N‐terminal glycine of a payload [190, 273]. The enzyme cleaves the LPXTG sequence between threonine (T) and glycine (G), exposing the threonine for nucleophilic attack by the payload's N‐terminal glycine via a thioacyl‐enzyme intermediate [273, 274, 275]. This method has been used to generate ADCs like Adcetris (brentuximab vedotin analog) and trastuzumab‐maytansine (Kadcyla analog) [269, 275, 276]. For these, the heavy (IgH) or light (IgL) chains of anti‐CD30 and anti‐HER2 antibodies were modified with an LPETG recognition sequence, while payloads (MMAE or maytansine) were functionalized with a pentaglycine linker [275, 276]. The resulting ADCs exhibited superior cytotoxicity compared to conventional counterparts, with trastuzumab‐maytansine achieving complete tumor regression in HER2+ SK‐BR‐3 xenograft models [275]. Despite its utility, Sortase A is restricted to N/C‐terminal modifications, prompting the adoption of self‐labelling tags (e.g., Halo‐tag, SNAP‐tag) for more flexible, homogenous conjugations [190, 277, 278, 279].

##### 1.3.2.2 Recombinant SNAP‐Tag‐Mediated Self‐Labeling

**FIGURE 3:**
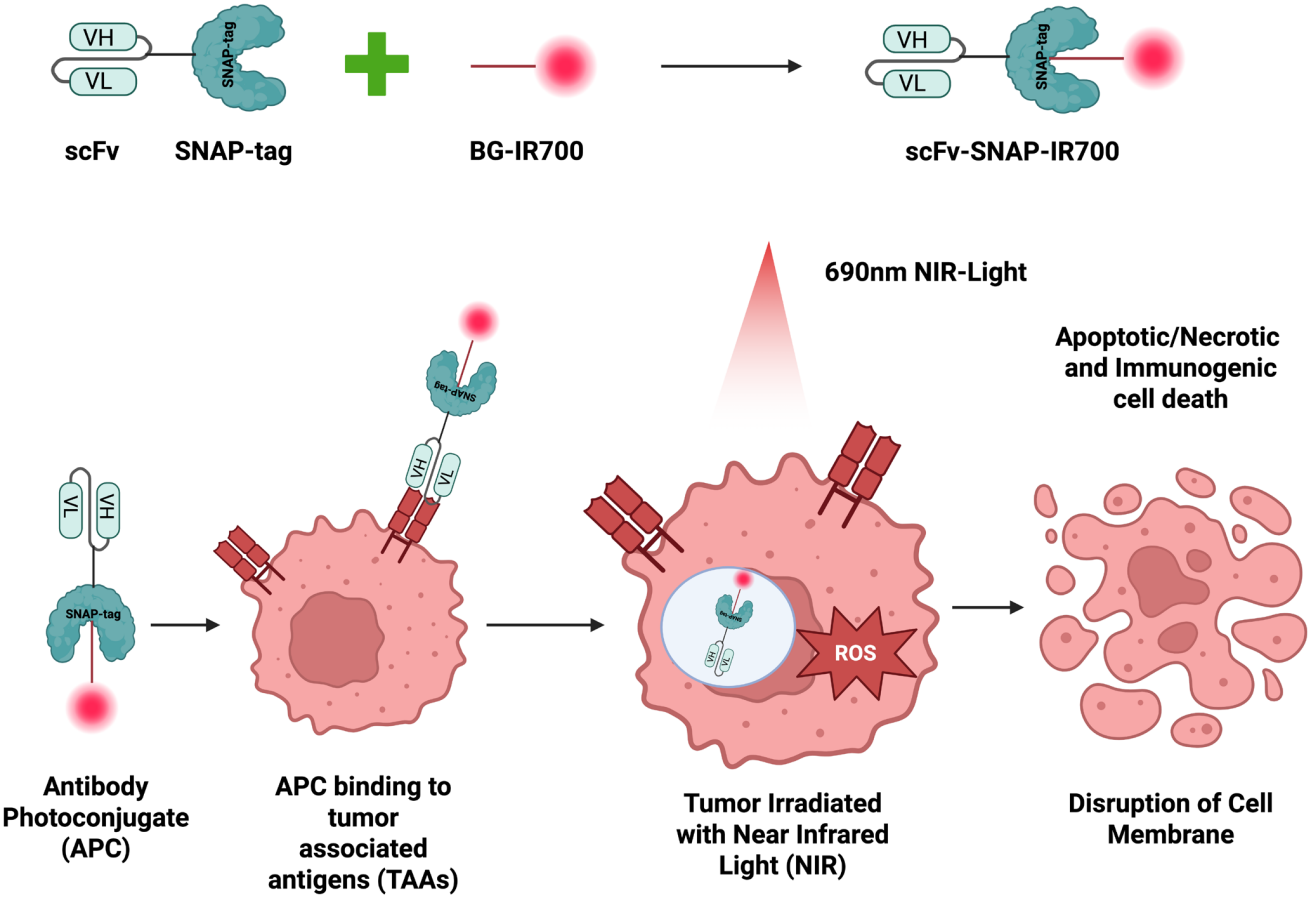
Photoimmunotherapy using SNAP‐tag‐based fusion protein. 1. Self‐labelling of SNAP‐tag fusion protein with benzylguanine‐modified near‐infrared dye IR700 (BG‐IR700) in a 1:1 stoichiometric ratio. 2. Binding of the scFv‐SNAP‐tag‐based photoimmunoconjugate to tumor‐associated antigen, 3. Illumination of the targeted tumor using near‐infrared light (690 nm), inducing the generation of reactive oxygen species (ROS), 4. leading to the activation of cell death mechanisms.

The Halo‐tag system utilizes an engineered haloalkane dehalogenase that forms irreversible covalent bonds with chlorohexane‐modified substrates (e.g., fluorescent dyes or affinity handles) under physiological conditioms [270, 271, 278]. Unlike its human‐derived counterpart SNAP‐tag (which originates from the 0 [6]‐alkylguanine‐DNA alkyltransferase repair protein), halo‐tag's non‐human origin makes it potentially more immunogenic [31, 280]. The SNAP‐tag system specifically reacts with benzyl guanine (BG)‐modified substrates through its human DNA repair protein scaffold [281, 282]. To be succinct, the unique and proprietary recombinant SNAP‐tag‐based antibody fusion protein format provides the following advantages in comparison to the state of the art: (1) reacts only and specifically with BG‐modified substrates; (2) conjugation is rapid and highly efficient under physiological conditions; (3) is self‐reactive (catalytic suicide enzyme function of AGT) and does not necessitate the addition of any further catalysts for conjugation; (4) can be expressed in a range of currently exploited expression systems (e.g., bacteria, yeast, or mammalian); (5) is the only currently self‐labeling tag of human origin with strong implications related to expected immunogenicity; and (6) generates homogenous conjugates based on a 1:1 stoichiometric conjugation reaction. For the past decades, SNAP‐tag has been genetically fused to small‐size single‐chain antibody fragments (scFvs), thereby conferring superior tumor penetration compared to their full‐length mAbs counterparts [7, 9, 51, 280, 283, 284, 286]. Moreover, their conjugation with diverse benzylguanine‐modified substrates (BG‐Alexa Fluor488, BG‐Vista green, BG‐Alexa Fluor 647, BG‐IR700, and BG‐AURIF) has shown their ability to detect and kill tumor cells expressing target antigens specifically [7, 9, 51, 280, 283, 284, 285, 286]. By conjugating these scFv‐SNAP‐tag immunoconjugates with corresponding BG‐modified imaging agents (e.g., BG‐Alexa Fluor488), authors were able to visually detect and observe subsequent lysosomal internalization of the immunoconjugates within targeted melanoma, prostate, and TNBC tumor cells [7, 9, 51, 280, 283, 284]. Additionally, the fluorescently labeled scFv‐SNAP‐tag immunoconjugates have demonstrated their capacity to profile South African TNBC tumor biopsies for clinically relevant EGFR, CSPG4, and CD44 biomarker expression [7, 51, 283]. Furthermore, the potency of the MMAF‐conjugated scFv‐SNAP‐tag‐based fusion proteins was achieved using sub‐nanomolar IC50 values [7, 51, 286]. Besides this, scFv‐SNAP‐tag based photoimmunotheranostic agents were developed with the aim to minimize normal tissue toxicity while conserving similar therapeutic profiles [9, 280, 283, 284, 287]. The pharmacokinetic behavior of these SNAP photoimmunoconjugates using a preclinical mouse model was investigated and demonstrated to specifically and optimally accumulate in EGFR‐expressing tumor xenografts 10 h after injection, with complete renal clearance achieved at 72h [287]. Like their MMAF‐based immunoconjugates, these photoimmunoconjugates were able to exclusively destroy illuminated tumors using similar IC_50_ values through a therapeutic procedure known as PIT [9, 280, 283, 284] (Figure 3).

#### 1.3.3 Therapeutic Effects of Near‐Infrared PIT on TNBC

**FIGURE 4:**
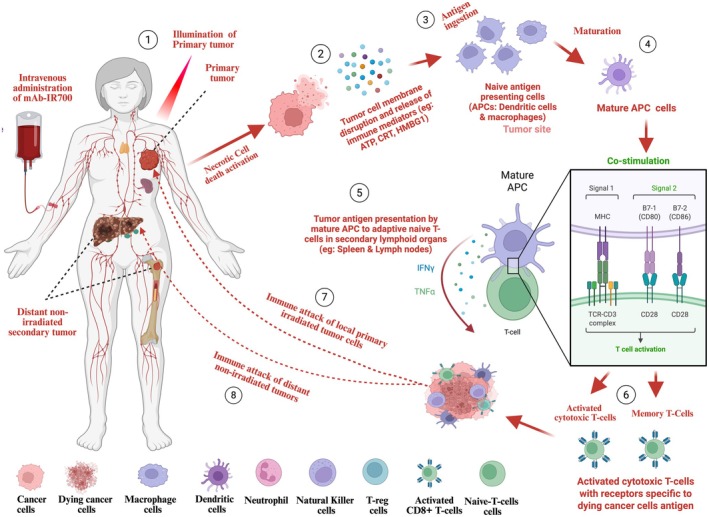
Photoimmunotherapy induced antitumor immunity and the abscopal effect. Illumination of the primary TNBC tumor leads to cancer cell membrane disruption and release of immunological signals, including heat shock proteins 70 (Hsp70) and 90 (Hsp90), calreticulin (CRT), adenosine triphosphate (ATP), and high‐mobility group box 1 (HMGB1) protein, activating nearby naïve innate immune antigen‐presenting cells (e.g., dendritic and macrophage cells), as well as those in the tumor microenvironment (TME). These APCs mature by engulfing cancer‐specific antigens released by dying cancer cells. Thereafter, the mature APCs present the tumor‐associated antigens to naïve T‐cells, thereby priming them to differentiate into cytotoxic T‐cells with receptors targeting the cancer cell antigens. The activated cytotoxic T‐cells then proliferate and attack both local irradiated and distant non‐irradiated tumor metastases. Photoimmunotherapy effectively functions as a vaccination, as it induces long‐lasting immune memory against cancer.

PIT is a therapy utilizing an antibody photoconjugate (APC) consisting of a recombinant antibody fragment conjugated to a light‐sensitive near‐infrared IR700 dye (NIR) known as PS [52, 63, 283, 288]. These APCs are nontoxic in their native administered form and are capable of specifically recognizing and binding to cell surface expressed TAAs through their recombinant antibody fragment moiety [9, 63, 289, 290]. Thereafter, the entire APC complex is internalized within the targeted cancer cells and only unleashes its phototoxic effects post‐light exposure (690 nm) through the generation of ROS, which ultimately cause tumor destruction through apoptotic, necrotic, and ICD (Table 1) [8, 35, 52, 61, 291, 292, 293, 294, 295]. PIT is inherently safe by virtue of requiring an extra step of light activation to exert its phototoxicity, thereby sparing normal tissues. Apart from that, PIT uses the excellent water‐soluble IR700 as PS, which has been demonstrated to lack cell membrane passive diffusion activities and possess great photostability with strong fluorescent signal properties [296, 297]. This NIR dye (IR700) optimally absorbs at 690 nm, a wavelength that deeply penetrates tumor tissues and is needed to maximize PS activation [296, 297]. PIT holds many therapeutic promises, with its recent clinical approval in Japan (2020) for treating HNSCC patients with advanced EGFR‐expressing tumors, which were unresponsive to radiotherapy, chemotherapy, and immune checkpoint blockade (ICB) therapies (e.g., anti‐PD‐1 or anti‐CTLA4) [290, 298]. Besides what precedes, PIT has the capacity to induce abscopal effects, characterized by the antitumor immune‐specific destruction of both primary irradiated and distant non‐irradiated tumor metastases when combined with ICB therapy (Figure 4) [63, 65, 299]. This antitumor immune augmenting effect of the PIT combination with ICB has extensively been reported against numerous breast cancer TAAs, including CD44, HER‐2, and EGFR receptors (Table 1) [8, 62, 63, 65, 300, 301]. Of note, mice that experienced complete tumor remission were able to resist subsequent tumor re‐challenge, thereby indicating the establishment of a long‐lasting systemic antitumor immune memory [63, 294, 302]. Likewise, Maruoka et al., 2020, Masaaki, and Glabman et al., 2024, have all demonstrated PIT's ability to reprogram the immunosuppressive TME into a responsive one through selective photodestruction of immune suppressive fibroblast activating protein (FAP)‐expressing cancer‐associated fibroblasts (CAFs) and CD25‐expressing regulatory T‐cells (NCT04305795, NCT05265013, jRCT2031220721) [63, 65, 303, 304, 305, 306]. By selectively killing FAP‐α‐expressing CAFs in TME, PIT was not only capable of inducing tumor regression, but simultaneously increasing the levels of IFN‐γ, TNF‐α, and IL‐2 in CD8+ tumor‐infiltrating lymphocytes and synergistically reverting tumor resistance in cells that were previously resistant to 5‐fluorouracil (5‐FU) chemoradiotherapy [303, 304, 305]. Additionally, Zhang et al., 2016 reported the significantly induced TNBC tumor growth delay when selectively targeting immunosuppressive αCD26‐expressing M2 macrophages found in sorafenib‐resistant 4 T1 tumors [307]. These results were corroborated by Barnett et al., 2022 and Fukushima et al., 2022 findings, showing PIT efficacy in specifically depleting CD11b^+^Gr1^+^ myeloid‐derived suppressor cells (MDSCs) and intercellular adhesion molecule‐1‐expressing cells in TNBC tumors [308, 309]. Besides what precedes, antitumor augmenting effects could be achieved by optimizing the APC conjugate light dosing treatment schedules on TNBC tumors [40, 310]. In this regard, both Ogata et al. and Nagaya et al. reported significant tumor regression and prolonged survival (above 200 days) in EGFR‐expressing treated TNBC mice, which received fractionated PIT in comparison to their single PIT dose‐treated counterparts [40, 310]. These long‐lasting responses could be attributed to PIT‐induced increases in the permeability of tumor vessels, thereby enabling continuous irradiated tumor perfusion with circulating inactivated APC through a phenomenon defined as the super enhanced permeability and retention (SUPR) effect [311, 312]. This marked PIT‐induced enhanced permeability and tumor‐specific drug retention effect was reported by Sano et al., 2013, demonstrating the positive correlation between the 24‐fold increased concentration of daunorubicin‐loaded liposomes in PIT irradiated EGFR‐expressing tumors and the extended survival post‐treatment [311]. Therefore, PIT can greatly augment the delivery of APCs or nanosized agents within irradiated tumors and thus holds promise to enhance cancer therapeutic treatment efficacies. The photoimmunotheranostic effects against CD44‐expressing TNBC tumors have previously been reported using xenograft orthotopic TNBC murine models, whereby the putative therapy was able to selectively destroy CD44‐expressing tumors, thus enhancing tumor‐free survival [8, 65, 291]. Recently, the selective detection and killing of αCD44, chondroitin sulfate proteoglycan‐4 (CSPG4), epithelial cell adhesion molecule (EPCAM), and EGFR‐expressing TNBC cancer cells using recombinant scFv‐SNAP‐tag‐based fusion proteins conjugated to IR700 dye was reported [9, 283]. This SNAP‐tag‐based PIT activity was further exemplified by Hussain et al. showing the selective detection and killing of EGFR‐expressing TNBC using scFv‐425‐SNAP‐tag conjugated (derived from Cetuximab mAb) to chlorine6 as PS [284]. The potency of PIT has been demonstrated in many cancers including but not limited to pancreatic, epithelioid sarcoma, lung, colorectal [65, 280, 284, 299, 300, 313, 314]. For instance, Hiroshima et al., 2015 reported the PIT tumor bed sterilizing effects (removal of post‐surgery residual tumor cells), precluding relapses after treating CEA‐expressing patient‐derived orthotopic pancreatic tumor xenografts [314]. These compelling and promising preclinical studies paved the way for the initiation of numerous clinical trials targeting CD25 (jRCT2031220721) and EGFR (NCT04305795) in combination or not with pembrolizumab immunotherapy (anti‐PD‐1) [63, 65, 306, 315] (Figure 4).

**TABLE 1:** Target molecules and photoimmunotherapeutic treatment.

Cancer type	Target	Photoimmunoconjugate	Survival benefits	Cell line	References
Triple‐negative breast cancer	ICAM‐1	Anti‐ICAM‐1‐IR700	Selectively depleted targeted cells with improved survival	MDA‐MB‐468luc and MDA‐MB‐231	[309]
Triple‐negative breast cancer	GR‐1	Anti‐GR‐1‐IR700	Specific killing of MDSCs	4T1‐luc and CD11b + Gr1+ MDSCs	[308]
Triple‐negative breast cancer	CD44	Anti‐CD44‐IR700	Destruction of targeted cells and tumor vasculature	MDA‐MB‐231 and BT‐474 cells	[8]
Triple‐negative breast cancer	EGFR	Anti‐EGFR‐IR700 (Cetuximab mAb)	Repeated PIT treatment schedules were associated with improved efficacy and longer survival	MDA‐MB‐468 and MDA‐MB‐231	[40]
Triple‐negative breast cancer	CD206	Anti‐CD206‐IR700	Specific killing of CD206 expressing M2 macrophages, reduction of TNBC lung metastasis and suppression of sorafenib resistance in treated TNBC tumors	4 T1 and CD206 Macrophages	[307]
Breast cancer	HER‐2	Anti‐HER2‐IR700 (Trastuzumab mAb)	Selective destruction of targeted tumors	BT‐474, SK‐BR‐3 and BT‐20 cells	[301]
Triple‐negative breast cancer	EGFR	Anti‐EGFR‐IR700	Selective destruction of targeted tumors	MDA‐MB‐468luc	[318]
Colorectal, lung and triple‐negative breast cancer	Fibroblast activation protein‐alpha (FAP)	Anti‐FAP‐IR700	Effectively depleted FAP^+^ CAFs and FAP^+^ myeloid cells Reduced lung and bone marrow metastases of TNBC tumors	4T1, LL2, MC38 and cancer associated fibroblast (CAFs) cells	[305]
Triple‐negative breast cancer	EGFR	Anti‐EGFR‐IR700 (Panitumumab mAb)	PIT combination with NIR releasing compound demonstrated superior antitumor activities than monotherapies	MDA‐MB‐468 cells	[319]
Triple‐negative breast cancer	CD44	CD44 (scFv)‐SNAP‐IR700	Specifically bind to cancer cells and TNBC FFPE TNBC tumor biopsies and kill targeted cells	MCF7, MDA‐MB‐468, MDA‐MB‐231 and Hs578 Cells	[283]
Triple‐negative breast cancer	EpCAM, CSPG4 and EGFR	Anti‐EpCAM (scFv)‐SNAP‐IR700 Anti‐CSPG4 (scFv)‐SNAP‐IR700 Anti‐EGFR (scFv)‐SNAP‐IR700 (Cetuximab)	Specifically detect target cells, bind to TNBC biopsies and kill targeted cells	MCF7, MDA‐MB‐468, MDA‐MB‐231, MDA‐MB‐453 and Hs578 Cells	[9]
Triple‐negative breast cancer cells	EGFR	Anti‐EGFR (scFv)‐SNAP‐Chlorin e6 (Cetuximab)	Specifically detect target cells, bind to TNBC biopsies and kill targeted cells	MDA‐MB‐468 and MDA‐MB‐231 cells	[284]
Triple‐negative breast cancer cells	EGFR	Anti‐EGFR‐IR700	Repeated PIT exposure promoted re‐perfusion of treated tumor and enhanced antitumor activity and survival	MDA‐MB‐468 cells	[310]
Unresectable locally advanced or recurrent head and neck cancer	EGFR	Anti‐EGFR‐IR700 (ASP‐1929‐402)	Post‐market clinical trial	N/A	(jRCT2041230050)
Advanced or recurrent solid tumors with liver metastases	CD25	Anti‐CD25‐IR700 (RM‐1995‐102)	Phase‐I clinical trial	N/A	(jRCT2031220721)
Recurrent or metastatic head and neck squamous cell carcinoma or locally advanced or metastatic cutaneous squamous cell carcinoma	EGFR	Anti‐EGFR‐IR700 (ASP‐1929‐181)	Phase 1b/2	N/A	(NCT04305795)

#### 1.3.4 Outcomes From Recent PIT Clinical Trials

The first clinical study to investigate the use of PIT for the treatment of cancer was conducted in the United States in 2015 on patients with recurrent HNSCC [290]. About 65% of patients with advanced HNSCC experience locoregional recurrence of HNSCC following standard‐of‐care therapy (surgery, radiation therapy, chemotherapy, or targeted therapy with cetuximab). These patients are often associated with facial disfigurement and impairment in speech, swallowing, and breathing as a consequence of the disease and treatment received [316]. Given the very poor prognosis associated with standard‐of‐care therapy for rHNSCC, there has been an urgent need for new treatment approaches that can provide locoregional control of rHNSCC without debilitating treatment outcomes. In the above phase 1/2a clinical trial, the study drug RM‐1929 was a drug‐device combination on the Illuminox platform, consisting of the NIR‐dye IR700 conjugated to cetuximab (EGFR‐mAb) and a laser device combination.

The key outcomes from the study of RM‐1929 were documented in two parts. Part 1, which aimed at determining the maximum tolerable dose (dose‐finding study), recruited nine patients with unresectable and locally recurrent HNSCC. These patients (3 in each dosing cohort) received one cycle of 160, 320, or 640 mg/m^2^ of RM‐1929 intravenously and NIR‐light illumination of the tumor with a wavelength of 690 nm at 24 ± 4 h after RM‐1929 administration. In all cohorts, RM‐1929 PIT was well tolerated at all doses tested, with no dose‐limiting toxicity observed. Due to the expression of EGFR in the skin tissue, 2 of the 9 patients reported low‐grade photosensitivity. Other treatment‐related side effects of RM‐1929 reported included oral pain and tumor pain, which were also moderate. One of the promising outcomes from the part 1 study was that 1 of 3 patients who received 640 mg/m^2^ of RM‐1929 achieved complete remission, while 7 of the 9 patients achieved disease control following just a single cycle of RM‐1929 PIT. Based on this promising result, 640 mg/m^2^ was selected as the recommended dose for part 2, which aimed to study the safety and efficacy of repeated cycles of RM‐1929 treatment in patients who have failed to respond to multiple prior therapies. In part 2, 30 patients with HNSCC were recruited across different centers and received 1–4 cycles of 640 mg/m^2^ RM‐1929 at intervals of 4 to 8 weeks. In this treatment cohort, 4 patients (13.3%) achieved complete remission, 9 patients (30.0%) achieved partial remission, and 24 patients (80.0%) showed disease control. Survival rates among patients were 49.8% (number at risk, *n* = 14) at 12 months, 29.1% (number at risk, *n* = 6) at 18 months, and 27.9% (number at risk, *n* = 9) at 24 months. The results showed that RM‐1929 PIT produced a longer median overall survival (9.3 months) when compared to second‐line systemic treatments (median overall survival range of 6–8 months) [290]. Also, four RM‐1929 treated patients in the part 2 cohort remained treatment‐free and alive up to 3 years in a long‐term follow‐up study. These results highlight the potential of PIT to provide significant benefits in patients with rHNSCC who do not respond or are not candidates for salvage treatments.

Of note is that while PIT does confer the advantage of tumor‐specific cell killing, the safety and efficacy study of RM‐1929 showed different types of treatment‐induced adverse effects that can be associated with PIT in humans. For example, 13 patients in the RM‐1929 part 2 study had serious adverse effects after receiving RM‐1929 PIT, of which 3 were considered treatment related. Three patients with serious adverse effects (tumor hemorrhage, arterial hemorrhage, and pneumonia), which led to deaths, were also reported for 3 patients who died between 19 and 32 days after receiving the last treatment. These deaths were considered by investigators to be caused primarily by tumor response to treatment or tumor encroachment on vessels. Essentially, in patients with tumors invading large blood vessels, PIT can cause rapid tumor collapse, which can lead to fatal bleeding in these patients. As such, patients with tumor invasion of large blood vessels might be at risk of arterial rupture and might not be eligible for PIT [317].

In the year 2017, a similar phase 1 clinical study of RM‐1929 was carried out in Japan in HNSCC patients who would not benefit from or failed to respond to prior treatment with surgery, radiotherapy, or chemotherapy [298]. The study recruited three patients in a single‐center and open‐label study who received a single 640 mg/m^2^ cycle of RM‐1929 followed by light illumination at 24 h thereafter. The outcome of this human study was likewise encouraging, as 2 of the 3 patients achieved partial remission. This resulted in the Japanese Ministry of Health, Labour and Welfare approving the use of cetuximab‐IR700 (Alluminox) to treat unresectable locally advanced or recurrent head and neck cancer in the year 2020. On the other hand, a fast‐tracked global Phase 3 study of RM‐1929 (ASP‐1929) commenced in 2019 and is currently ongoing (NCT03769506) in patients who have failed at least two lines of therapy across different centers in the United States, Taiwan, Spain, Japan, Greece, and Belgium. Other clinical trials being conducted with ASP‐1929 include a U.S. phase 1b/2 clinical trial for recurrent HNSCC and cutaneous squamous cell carcinoma (CUSCC) using combination therapy with anti‐PD1 (NCT04305795) and a phase 2 clinical trial with fluorescence imaging for operable primary or recurrent head and neck or CUSCC (NCT05182866). Furthermore, a phase‐I dose‐escalation clinical study using anti‐CD25‐IR700 conjugate (RM‐1995) alone or in combination with pembrolizumab was initiated to treat patients with advanced or recurrent solid tumors with one or more hepatic metastatic lesions (jRCT2031220721). Understanding the immune‐modulating effects of PIT combined with immunotherapy is critical for treating patients resistant to standard therapies, including immune checkpoint inhibitors (ICIs). While preclinical studies strongly support PIT's ability to induce antitumor immunity, clinical evidence remains limited [63, 65, 315]. Recently, Hiromasa et al. (2024) analyzed blood samples from PIT‐treated patients, revealing post‐treatment increases in DAMPs (HMGB1, HsP70) and chemokines (CCL3, CCL4) [320]. Patients with low neutrophil‐to‐lymphocyte ratios (NLR) showed better responses and survival than those with high NLR or low DAMPs levels [320]. However, since this study relied solely on peripheral blood, further research is needed to assess tumor‐specific immune changes. These findings highlight the importance of localized immune profiling in future trials, such as the ongoing phase‐3 study by Mainakas et al. which evaluates PIT combined with pembrolizumab in recurrent head and neck squamous cell carcinoma against standard‐of‐care therapy [321]. Given the recalcitrant nature of TNBC tumors to current therapies and their propensity to metastasize in the brain, bone, and liver tissues, it becomes evident that PIT is a promising, safe, minimally invasive therapy with predictable reduced side effects, as clinically reported by their non‐targeted PDT [54]. PIT has demonstrated efficacy against chemotherapy, radiotherapy, and immunotherapy‐resistant tumors, including maxillary sinus, cervical, vulvar, vaginal, oropharyngeal, and liver metastases, for which complete responses were documented (see Table 2).

**TABLE 2:** Clinical application of PIT and therapeutic outcomes.

Cancer type	Drug name	Phase	Patients characteristic	Results	References
Recurrent head‐and‐neck squamous cell carcinoma (rHNSCC)	RM‐1929 (anti‐EGFR–IR700)	Phase‐1/2b	Unresponsive to platinum‐based chemotherapy treatment	Averse events unrelated to photoimmunotherapy Objective response rate (ORR: 43.3%) Overall survival (OS: 9.30 months)	[290]
Recurrent laryngeal cancer cervical	RM‐1929 (anti‐EGFR–IR700)	Case report	Underwent surgery and radiotherapy	Patient experienced partial response after first PIT session Disease progression after second PIT session	[322]
Recurrent oropharyngeal squamous cell carcinoma at tongue base	RM‐1929 (anti‐EGFR–IR700)	Case report	Unresponsive to chemotherapy and radiotherapy	Complete response (CR) with no postoperative functional impairment	[323]
Recurrent nasopharyngeal squamous cell carcinoma	RM‐1929 (anti‐EGFR–IR700)	Case report	Unresponsive to radiotherapy	CR for more than a year post‐treatment	[324]
Recurrent oropharyngeal and cervical metastasis	RM‐1929 (anti‐EGFR–IR700)	Case report	Radiotherapy, boron neutron capture therapy, surgery	CR three months post‐treatment	[325]
Recurrent head‐and‐neck cancer	RM‐1929 (anti‐EGFR–IR700) + Pembrolizumab (anti‐PD‐1)	Case report	Unresponsive to radiotherapy and PIT	Complete destruction of recurrent lesions 2 months post‐PD‐1 treatment CR for more than a year	[326]
Recurrent locally advanced and metastatic head‐and‐neck squamous cell carcinoma (r/m‐HNSCC)	RM‐1929 (anti‐EGFR–IR700)	Phase‐1/2b	N/A	ORR: 33.3% OS (more than 24 months): 52.4% CR: 22.2% Partial response (PR): 11.1%	[327]
Head‐and‐neck squamous cell carcinoma (HNSCC)	RM‐1929 (anti‐EGFR–IR700)	Case report of 7 patients	N/A	Patients with low neutrophil‐to‐lymphocyte ratio (NLR) pre‐PIT treatments were associated with better therapeutic response and survival rates Increase DAMP release post‐PIT correlates with better therapeutic response Baseline NLR predict patient response to PIT.	[320]
Unresectable recurrent maxillary sinus cancer employing	RM‐1929 (anti‐EGFR–IR700) + Surgery + CT guidance	Case report	Unresponsive to chemoradiotherapy and immunotherapy	Complete tumor necrosis and destruction with no adverse events and pain	[328]
Advanced or recurrent solid tumor with liver metastatsis metastases	RM1995 (anti‐CD25‐IR700) alone or in combination with Pembrolizumab (anti‐PD‐1)	Phase‐1	Unresponsive to standard of care	Currently recruiting	jRCT2031220721
Advanced gastric cancer	RM‐1929 (anti‐EGFR–IR700) + Nivolumab (anti‐PD‐1)	Phase‐1b	Unresponsive to chemotherapy and immunotherapy	No dose‐limiting toxicity (DLT) observed Combining endoscopic PIT with nivolumab is safe and feasible	[329]
Recurrent vulvar, vaginal, cervical cancer	RM‐1929 (anti‐EGFR–IR700)	Phase‐2	Unresponsive to radiotherapy	Study initiated	jRCT2011240034
Esophageal cancer	RM‐1929 (anti‐EGFR–IR700)	Phase‐1b/2	N/A	Study initiated	jRCT2080224666
Locally advanced and locally recurrent head‐and‐neck carcinoma (LA‐LR‐HNC)	RM‐1929 (anti‐EGFR–IR700)		Unresponsive to chemoradiotherapy	ORR: 89% Good tumor control and patient quality of life (QOL)	[330]

To enhance the efficacy of PIT, novel APCs targeting clinically relevant cancer biomarkers must be developed and evaluated in preclinical animal models. For instance, Takashi et al. demonstrated PIT's ability to detect and eliminate pancreatic tumors overexpressing TROP2, a promising clinically relevant target given the FDA approval of Sacituzumab govitecan, an anti‐TROP2 ADC conjugated to SN8 (topoisomerase inhibitor) for treating metastatic TNBC [331, 332]. Repurposing this ADC by replacing SN38 with IR700 could yield a potent PIT‐based therapy for light‐accessible TNBC. Similarly, SGN‐CD228A, an auristatin‐E‐based ADC, has shown efficacy against melanotransferrin receptor‐positive cancers (eg., melanoma, colorectal, pancreatic, squamous non‐small cell lung cancer, and breast cancer), with ongoing clinical evaluation (NCT04042480). Supporting this approach, Magagoum et al. reported that SNAP‐tag‐based APCs through PIT were capable of selectively targeting melanotransferrin‐overexpressing melanoma and TNBC nanomolar IC_50_ concentrations [281]. Further improvement of PIT can be achieved by identifying the most immunogenic light dose or treatment schedule, which will maximally activate ICD. For instance, Hsu et al., 2022 demonstrated the antitumor immune‐augmenting effects of PIT in combination with anti‐PD‐1 immunotherapy, which increased the complete responders’ rate from 7.7% (1 out of 13 mice: PIT only) to 50% (7 out of 14 mice: PIT + Immunotherapy) [63]. Likewise, Yasurisho et al. 2demonstrated that combining PIT targeting CD44 with interleukin‐15 (IL‐15), a cytokine that activates natural killer cells, enhances antitumor immunity, leading to stronger tumor growth suppression, prolonged survival, and increased CD8+ T‐cell infiltration compared to monotherapies [333]. This immunomodulatory effect of PIT was further supported by Hiromasa et al., 2024, who showed that low NLR and high DAMPs release could serve as predictive biomarkers for treatment response [320]. Given the limited understanding of PIT's antitumor immune mechanisms in clinical settings, along with observed durable responses when combined with ICIs, future clinical trials should incorporate long‐term patient monitoring to correlate immune profiling, treatment regimens, and dosing with therapeutic outcomes, since antitumor immunity has been demonstrated to exert a vaccination effect precluding the development of metastases, hence tumor recurrence.

#### 1.3.5 Improving PIT Using NPs

NPs are structures 1–100 nm in size, either organic or inorganic, that have been shown to enhance site‐specific drug delivery. They can improve the biodistribution and uptake of surface‐immobilized or encapsulated drugs [334] due to their biocompatibility and hydrophilic nature, which, similar to PEG, improves the solubility and consequently the pharmacokinetics of these drugs [335]. NP surfaces are typically easy to functionalize with targeting ligands, such as antibodies or antibody fragments, to achieve site‐specific drug delivery [336]. Further functionalization, for example, with PEG, is also often performed to increase NP biocompatibility such that they provide greater stealthing for the drugs they carry, which results in longer circulation times [337, 338, 339]. Moreover, the inherent biocompatibility of many NP formats, as well as the enhanced biocompatibility conferred through functionalization, helps NPs mimic biomolecules, which enables them to go undetected by the host immune system and avoid interference through degradation and clearance [336, 340]. In cancer treatment in particular, the small size of NPs reportedly facilitates tumor localization via the enhanced permeability and retention effect, whereby the leaky vasculature of the tumor microenvironment results in preferential accumulation in this tissue [82]. Additionally, NPs exhibit large area‐to‐volume ratios (aspect ratio), allowing them to carry high drug loads, which reduces the minimal effective dose and increases the therapeutic index [341, 342].

Studies have shown that the incorporation of nanotechnology into PDT can augment the phototoxic effects of the therapy in vitro by increasing the uptake and intracellular concentration of a photosensitizer using gold [343, 344, 345], gold‐silver alloyed bimetallics [346], gold dendrimers [347, 348], silver [349], silica [350, 351], carboxylated poly (amido‐amine) [352], cubosomes [353], and liposomes [354], among others. Various NP materials have been assessed to identify and manipulate them to have optimal optical and physiochemical properties [355, 356]. The hydrophobic nature of most photosensitisers can limit cellular drug accumulation of these compounds, but NPs take advantage of the EPR effect [357, 358], resultantly increasing cellular uptake [359]. While NPs cannot extravasate the thicker epithelial cells of normal healthy blood vessels, the conditions of the tumor microenvironment lead to leaky vasculature, allowing NPs to accumulate preferentially in the tumor tissue [360]. Stealthing of NPs can be further enhanced through PEGylation, and this has been shown to not only improve half‐life but also reduce immunogenicity and delay host immune responses [339]. Furthermore, Xiaopin et al., 2016, reported the phototoxic and antitumor immune augmenting effects of zinc pyrophosphate NPs (loaded with PS pyrolipid (ZnP@pyro)) based PDT when combined with anti‐PDL‐1 immunotherapy against 4T1 TNBC tumors [361, 362]. These results were corroborated by Zhen et al. showing the enhanced tumor‐specific T‐cell infiltration and 4 T1 tumor regression following the targeted destruction of FAP‐alpha‐expressing CAFs using ferritin NPs conjugated to FAP‐alpha‐scFv [363].

## 2 Limitations of PIT

The scattergun effect associated with PDT may have resulted in the creation of PIT, but several challenges remain despite its targeted action and ability to induce ICD. Widespread clinical adoption of PIT would necessitate improving the stability of the PSs while reducing their risk of photobleaching to ensure treatment consistency [364]. Additionally, the need for specialized light‐delivery systems and imaging technologies, alongside the costs of PSs, potentially limiting scalability and patient accessibility, is debated [365]. To exacerbate the situation, external light delivery may not be suitable in the case of hypoxic tumor microenvironments, whereby limited light penetration may hinder the generation of oxygen‐dependent reactive species [364]. As an alternative to PIT, bispecific antibodies (bsAbs) may be postulated for their systemic delivery and engagement with immune effector cells to target tumors without the need for light activation [366, 367]. Indeed, several bsAbs, such as ROR1/CD3 [368] and BNT327, are currently in preclinical or early clinical trial stages for TNBC. While preliminary data look promising, bsAbs can come with their own risks, including cytokine release syndrome (CRS) and neurotoxicity (ICANS) [369]. Therefore, while PIT offers a unique mechanism of action, which must be further optimized, its overarching benefit must be weighed against such alternatives like bsAbs in terms of feasibility, large‐scale manufacturing, and patient safety.

## 3 Conclusions

PIT is a promising therapeutic modality for the treatment of various cancers, particularly those readily accessible to therapeutic light, such as melanoma, cervical, and TNBC tumors. By targeting cancer cells, immunosuppressive cells, or both, in a light‐controlled manner, it has substantially induced superior antitumor activities with minimal side effects as opposed to traditional chemo and radiotherapy. When combined with immunoadjuvant therapies, it can destroy local and disseminated metastatic tumor lesions while establishing antitumor immune memory responses, precluding the recurrence of any residual tumor cells. With significant research efforts in optimizing these combined approaches, PIT is poised to replace conventional therapies and become the mainstay first‐line therapy for treating TNBC tumors.

## Author Contributions

F.A.N.B., N.M., S.B., and W.N. conceptualization, review, and writing of the original draft. F.A.N.B., N.M., N.E.T.C., Z.M., S.Y., O.A.A., K.A.S., G.M.M., F.E.C., S.S., W.N., and S.B. contributed to review, writing, and editing. F.A.N.B. and N.M.: visualization, validation, formal analysis, and data curation. All authors read and approved the final version of the manuscript.

## Conflicts of Interest

The authors declare no conflicts of interest.

## Data Availability

Data sharing not applicable to this article as no datasets were generated or analyzed during the current study.
